# Tracing and constraining anthropogenic aerosol iron fluxes to the North Atlantic Ocean using iron isotopes

**DOI:** 10.1038/s41467-019-10457-w

**Published:** 2019-06-14

**Authors:** Tim M. Conway, Douglas S. Hamilton, Rachel U. Shelley, Ana M. Aguilar-Islas, William M. Landing, Natalie M. Mahowald, Seth G. John

**Affiliations:** 1 College of Marine Science and School of Geosciences, University of South Florida, Tampa, FL USA; 2 Department of Earth and Atmospheric Sciences, Atkinson Center for a Sustainable Future, Cornell University, Ithaca, NY USA; 3 Department of Earth, Ocean and Atmospheric Science, Florida State University, Tallahassee, FL USA; 4 College of Fisheries and Ocean Sciences, University of Alaska Fairbanks, Fairbanks, AK USA; 5 Department of Earth Sciences, University of Southern California, Los Angeles, CA 90089 USA

**Keywords:** Element cycles, Atmospheric chemistry, Marine chemistry

## Abstract

Atmospheric dust is an important source of the micronutrient Fe to the oceans. Although relatively insoluble mineral Fe is assumed to be the most important component of dust, a relatively small yet highly soluble anthropogenic component may also be significant. However, quantifying the importance of anthropogenic Fe to the global oceans requires a tracer which can be used to identify and constrain anthropogenic aerosols in situ. Here, we present Fe isotope (δ^56^Fe) data from North Atlantic aerosol samples from the GEOTRACES GA03 section. While soluble aerosol samples collected near the Sahara have near-crustal δ^56^Fe, soluble aerosols from near North America and Europe instead have remarkably fractionated δ^56^Fe values (as light as −1.6‰). Here, we use these observations to fingerprint anthropogenic combustion sources, and to refine aerosol deposition modeling. We show that soluble anthropogenic aerosol Fe flux to the global surface oceans is highly likely to be underestimated, even in the dusty North Atlantic.

## Abstract (web summary)

The relative importance of crustal vs. anthropogenic dust deposition for iron cycling in the surface ocean is unclear. Based on analysis of iron isotope data from North Atlantic aerosol samples, the authors can reveal the relative importance of anthropogenic iron emissions and its impact on marine biogeochemistry.

## Introduction

Iron (Fe) is an important marine micronutrient, thought to be acting as the principal control on phytoplankton growth and carbon export in up to a third of the world’s oceans^1,2^, as well as influencing global distribution of nitrogen fixation, due to the high Fe requirement of nitrogen-fixing diazotrophs^3^. However, the low solubility of Fe in seawater means that dissolved Fe concentrations are often vanishingly small away from Fe sources^4^. Deposition of Fe-bearing atmospheric dust, while highly episodic and regionally variable, may be the most important source of new Fe to the surface oceans in some Fe-limited regions^1,5^, and is especially important in the North Atlantic due to the close proximity to the Saharan dust source^6–8^. Consequently, changes in the deposition, composition, and distribution of aerosol Fe supply has the potential to dramatically influence biogeochemical cycles and the carbon cycle at the global scale, especially across climatic transitions such as glacial-interglacial change^9–11^. While natural desert dust from arid regions around the world has long been considered a globally important source of atmospheric Fe to the oceans over multiple timescales, in recent years, a number of observational and modeling studies have suggested that the release of Fe during combustion (including anthropogenic industrial sources, biofuel or fossil fuel burning, and global biomass burning) may also be an important supply of soluble Fe to the surface oceans^12–17^.

Disparate lines of evidence have previously suggested an important role for anthropogenic aerosol Fe, though the magnitude of this flux is currently poorly characterized. Anthropogenically produced aerosols, as fingerprinted either from air mass back-trajectory analysis or the presence of anthropogenically sourced elements (e.g. Ni and Pb from coal or fly ash or gasoline and V from heavy oil), have been suggested to contain relatively highly soluble Fe compared with mineral dust^14,15^. For example, high Fe solubilities (up to 19%) in marine aerosols collected near Bermuda have been linked to anthropogenic processes^14,15^, while mineral Fe within natural desert dust typically has very low solubility (1%)^18^. Although such studies demonstrate that aerosols can carry large amounts of soluble Fe, it is not possible to determine whether solubility is directly related to the presence of an anthropogenic component or whether Fe solubility has been enhanced due to physical and/or chemical atmospheric processing. For example, several workers have suggested that under low dust loading, or far from source, or under variable composition, aerosol solubility is enhanced from <1% up to 10% or even greater^19–21^. The challenges of determining a representative Fe solubility under laboratory conditions also complicates efforts. Overall, these complications lead to a large range in estimated aerosol Fe solubility from <1 to 95%^20,22^. Furthermore, measurements of Fe solubility in preindustrial (and thus truly natural) Last Glacial Maximum ice-core dust ranged from <1 to 40%^23^, suggesting that high Fe solubilities are not purely diagnostic of the presence of anthropogenic Fe.

Another approach to evaluating the biogeochemical importance of anthropogenic Fe deposited to the oceans is to use global aerosol deposition models, which resolve iron aerosol. Currently, modeling suggests that a small but highly soluble flux of combustion-sourced Fe has a major impact on the total estimated atmospheric soluble Fe delivery to the oceans worldwide^24,25^. Such models have even gone so far as to suggest that anthropogenic Fe may dominate soluble aerosol Fe deposition in some regions of the globe, especially to the ocean in the Southern Hemisphere^26–28^. However, a major uncertainty in extrapolating from observations to modeling global fluxes arises from uncertainties in the solubility of Fe from various natural and anthropogenic sources. Generally, modelers have assumed that Fe from mineral dust is very insoluble, using Fe solubilities of <2% (although this may not capture the observed range of spatial variability)^12,29,30^, while assuming much higher solubilities for anthropogenic Fe based on measurements from point sources^12^. However, both of these assumptions are subject to large uncertainties, principally because of the uncertainty over the causes of different Fe solubilities in observational studies. Similarly, using other elements as tracers for anthropogenic Fe requires them to be derived from the same source with a reproducible or representative ratio, which is challenging to quantify in a well-mixed atmosphere far from aerosol sources^31^. A quantitative evaluation of the global importance of anthropogenic Fe therefore requires a tracer which can be used to inform anthropogenic fluxes in situ.

The stable isotope ratio of Fe (δ^56^Fe) within aerosols may provide an additional way to constrain the relative importance of Fe sourced from natural dust versus other types of aerosol, provided there are resolvable and characterizable differences between natural and anthropogenic aerosol Fe. Natural Fe within bulk natural aerosol dust, and within material from dust source regions, has been characterized by a near-crustal δ^56^Fe value of +0.1‰ (relative to IRMM-014 standard)^32–36^. In addition, several recent studies have suggested that there is also an isotopically light marine aerosol Fe phase which may be linked to anthropogenic activity^34,37,38^. This attribution is supported by near-source measurements, which suggest that anthropogenic sources of aerosol Fe such as those emitted from combustion of gasoline can be highly fractionated (−3 to +0.3‰)^34,35,39,40^. Specifically, isotopically light δ^56^Fe values have been observed in bulk aerosol near anthropogenic sources such as a road tunnel at Hiroshima, Japan (−3 to +0.3‰)^40^. Similarly light δ^56^Fe values were measured in aerosols collected in the bulk phase (−2.01 to +0.23‰) and even lighter in simulated rain-water extractions (−3.91 to −1.87‰) at Hiroshima, Japan, over several months^34^, providing further evidence for a light δ^56^Fe anthropogenic endmember near combustion sources.

However, from these near-source measurements, which span a range of isotopic compositions and particle sizes, it is difficult to establish precisely what the δ^56^Fe signature of an anthropogenic endmember might be, were it to be measured in open-ocean marine aerosols. Insight for the latter is provided from measurements of the mean fine-fraction (<2 µm) bulk phase aerosols collected from in the winter at Bermuda (mean −0.1‰, as light as −0.5‰)^35^, and in the two samples of fine-fraction bulk phase aerosol particles from the North West Pacific (−1.2 and −1.7‰)^34^. Isotopically light bulk phase aerosol Fe has also been reported from Taiwan, linked to anthropogenic activity by high Fe solubility^37^. Together, these studies are indicative of the presence of an isotopically light Fe component within marine aerosols, meaning that aerosol δ^56^Fe values may allow us to distinguish between the presence of anthropogenic Fe from natural mineral Fe within an aerosol sample, provided we can constrain isotopic end members.

Here, we present new data for aerosol iron isotopes (δ^56^Fe) collected shipboard from the North Atlantic Ocean during the US GEOTRACES North Atlantic GA03 section. While bulk δ^56^Fe show some variability across the section, we observe a dramatic difference in soluble aerosol δ^56^Fe between samples collected from Saharan air masses (+0.1‰), and those collected from North American or European air masses, which show remarkably fractionated δ^56^Fe values (as light as −1.6‰). We attribute these Fe isotope signals to mineral dust and anthropogenic sources, respectively, demonstrating that the signature of anthropogenic combustion is visible at the basin scale even in the very dusty North Atlantic. By coupling these results to dust deposition modeling, we show that a reduction in natural dust source solubility and an increase in anthropogenic aerosol Fe supply to the region are needed to reproduce GA03 δ^56^Fe observations. Applying those model refinements at the global scale suggests that current parameterizations of dust deposition models may underestimate anthropogenic aerosol Fe fluxes to the global oceans.

## Results

### GA03 Fe isotope results

**Fig. 1:**
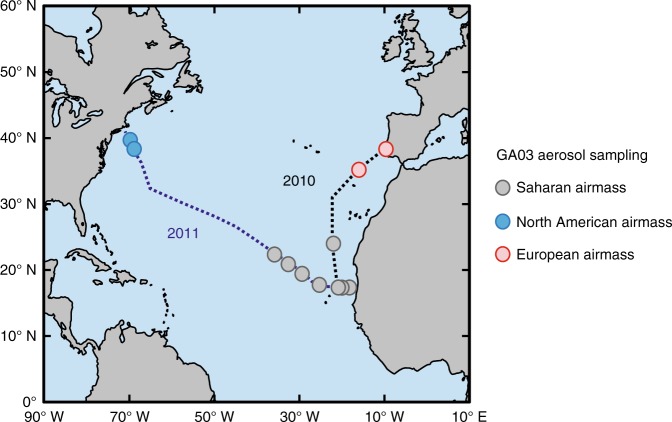
North Atlantic GA03 aerosol Fe isotope sampling locations. Aerosol samples are a subset of those collected on the cruise, and are characterized as Saharan (gray), North American (blue), or European (red), according to HYPSLIT air mass back-trajectory analysis^43^. 2010 and 2011 legs of the GA03 cruises (USGT10 and USGT11) are shown separately

We measured δ^56^Fe in total bulk (HNO_3_ and HF-digested) and soluble (water- and seawater-soluble) fractions of marine aerosols collected from above the North Atlantic Ocean during two GEOTRACES GA03 section cruises^41,42^ during winter 2010 and 2011. Based on published HYSPLIT back-trajectory modeling of air masses sampled on the cruises^43^, we chose a subset of samples (Fig. 1) from defined air masses, including areas strongly influenced by the mineral dust plume emanating from the Sahara desert (Saharan air masses) as well as regions near North America (North American air masses) and Europe (European air masses).

**Fig. 2:**
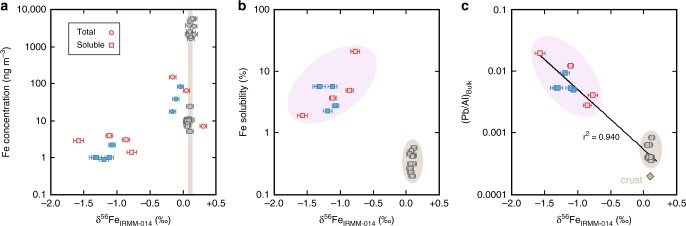
North Atlantic GA03 aerosol Fe isotope results. Water-soluble and bulk aerosol δ^56^Fe relative to **a** Fe concentration^43^, **b** Fe solubility^44,45,58^, or **c** bulk Pb/Al^43^. Aerosols from different air masses^43^ are separated by color: gray Saharan (2010 and 2011), red European (2010), and blue North American (2011). Error bars on δ^56^Fe are 2σ external error (0.05‰) or 2σ internal error (where larger). The brown vertical bar represents the δ^56^Fe signature of crustal source materials (0.07–0.14‰)^32^. Circles denote natural crustal (brown) or anthropogenic-sourced (purple) aerosol fields, based on this study. (Pb/Al)_Bulk_ and the crustal diamond in (**c**) are based on other sources^36,43,48^

We found that GA03 aerosols considered to be dominated by the Saharan dust plume were characterized by near-crustal δ^56^Fe in both the bulk and water-soluble phases (+0.12 ± 0.03‰ and +0.09 ± 0.02‰, respectively), confirming the observation that natural aerosol Fe is defined by near-crustal δ^56^Fe (Table 1; Fig. 2a^32,33^). Leaching of the same aerosol dust with surface seawater also yielded crustal values (+0.04‰ and +0.05‰; Supp. Data), consistent with the only other previous δ^56^Fe measurement for soluble Saharan aerosols^33^. However, in dramatic contrast to the Saharan-dominated aerosols, the aerosols collected from North American or European air masses showed pronounced fractionation toward lighter δ^56^Fe values (−0.8 to −1.6‰; mean −0.91‰) in the water-soluble phase (Table 1; Fig. 2a), the seawater-soluble phases (−1.45‰), and more-muted but similar fractionation towards lighter δ^56^Fe values in the bulk phase (−0.12 ± 0.06‰). While these lighter δ^56^Fe values could be caused by either a primary source signal or the result of kinetic isotope effects during partial dissolution or re-precipitation of Fe during leaching, the presence of lighter-than-crustal δ^56^Fe in the bulk aerosol, as well as similar observations in bulk phase marine aerosol from other studies^34,35^ means the isotopically light soluble phase cannot simply be attributed to differences in chemical behavior during processing or leaching. Instead, our data are indicative of at least two kinds of aerosol Fe present in the samples, one of which is natural and one of which we hypothesize to be anthropogenically sourced.

**Table 1:** Mean GA03 North Atlantic aerosol data (±1SD) for each air mass, as defined by back-trajectory modeling^43^

Air mass	Fe loading	Instantaneous Fe solubility	Mean bulk δ^56^Fe	Mean water-soluble δ^56^Fe	Mean (Pb/Al)_Bulk_
	(ng m^−3^)	%	‰_IRMM_	‰_IRMM_	
Saharan 2010	3000 ± 1300	0.4 + 0.2	+0.12 ± 0.03	+0.08 ± 0.03	0.0006 ± 0.0002
Saharan 2011	3100 ± 1600	0.3 ± 0.1	+0.13 ± 0.03	+0.09 ± 0.02	0.0004 ± <0.0001
Saharan	3000 + 1400	0.4 ± 0.1	+0.12 ± 0.03	+0.09 ± 0.02	0.0005 ± 0.0001
Euro. 2010	81 + 61	8.7 ± 7.8	−0.04 ± 0.03*	−1.20 ± 0.11	0.0096 ± 0.0077
N.Am. 2011	45 ± 32	3.6 ± 1.8	−0.16 ± 0.06	−1.08 ± 0.36	0.0057 ± 0.0034
Euro. & N.Am.	66 ± 51	6.0 ± 6.7	−0.12+0.06	−1.15 ± 0.24	0.0079 ± 0.0062
UCC	−	−	+0.09	−	0.0002

See Supplementary Data 1 for the full dataset. Saharan denotes aerosol from the Saharan air masses for both years, Euro & N.Am. denotes aerosols from either European or North American air masses. Fe solubility denotes instantaneous solubility in ultrapure water (see Methods). Fe loading, Fe solubility and (Pb/Al)_Bulk_ are reproduced from previous work^43–45, 58^, and UCC denotes typical upper continental crust composition^32, 48^. For *, only one sample was measured, so this is shown rather than the mean

### Comparison of Fe isotopes results with other aerosol parameters

The Saharan-dominated aerosols were characterized by much higher Fe loading than the non-Saharan samples (Table 1; Fig. 2a)^43^, an observation consistent with global aerosol models of highly soluble anthropogenic Fe mixing with a large contribution of very poorly soluble Fe from desert dust^15,26^. Indeed, that idea is in strong agreement with data in this study when water-soluble δ^56^Fe is plotted against Fe solubility^44,45^ (Fig. 2b); aerosol samples fall into two well-defined groups, either a natural dust field (δ^56^Fe +0.1‰, 0.2–0.5% soluble) or a hypothetical anthropogenic field (δ^56^Fe −0.5 to −1.6‰; 2–21% soluble). The isotopically light signature of anthropogenic Fe is thus largely masked in the bulk phase by the much greater presence of Saharan dust particles, but becomes dominant in the soluble phase due to the higher solubility of anthropogenic Fe^46,47^. Furthermore, in addition to the high Fe solubilities and light δ^56^Fe signatures, highly elevated (15–95×) bulk Pb/Al ratios (0.003–0.019)^43^ in the anthropogenic field (Fig. 2c; Table 1) compared with the upper continental crust Pb/Al (0.0002)^48^ are similarly indicative of anthropogenic pollution. This Pb/Al signature, together with elevated Ni/Al and V/Al over crustal in these samples^43,44^, while not definitive evidence that the light Fe is anthropogenic, provides further weight to the hypothesis that the light Fe-bearing aerosols within North American and European air masses are strongly influenced by anthropogenic activity.

## Discussion

Our data provide clear evidence for the presence of an isotopically light highly soluble Fe phase in marine aerosol over the Atlantic, which we suggest is linked to anthropogenic activity through chemical analyses. This scenario can be simply modeled using a two-component isotope mixing equation:

1 $$	{\mathrm{\delta }}^{{\mathrm{56}}}{\mathrm{Fe}}_{{\mathrm{soluble}}\;{\mathrm{aerosol}}} =\\ 	 \ \ \ \ \left( {{\it{f}}\!_{{\mathrm{dust}}} \times {\mathrm{\delta }}^{{\mathrm{56}}}{\mathrm{Fe}}_{{\mathrm{soluble}}\;{\mathrm{dust}}}} \right) + \left( {{\it{f}}\!_{{\mathrm{anthropogenic}}} \times {\mathrm{\delta }}^{{\mathrm{56}}}{\mathrm{Fe}}_{{\mathrm{soluble}}\;{\mathrm{anthropogenic}}}} \right)$$

which can be re-arranged to solve for the relative fractions of natural dust Fe (*f*_dust_) and anthropogenic aerosol Fe (*f*_anthropogenic_) in the soluble phase of the GA03 aerosol samples, by assigning fixed values for δ^56^Fe_soluble dust_ and δ^56^Fe_aoluble anthropogenic_, based on appropriate assumptions, and using the measured isotope ratio in each soluble aerosol sample (δ^56^Fe_soluble aerosol_). For this simple mixing model, we assigned +0.09‰ as the δ^56^Fe_dust_ endmember, which is the crustal Fe isotopic composition^32^ (confirmed for Saharan soluble aerosol in this study), and −1.6‰ as the anthropogenic aerosol endmember, based on data from this study and the literature (see Methods for further discussion of endmember choice). Using these assigned end members, geochemical modeling suggests that the European and North American soluble aerosols are characterized by ~50–100% anthropogenic Fe, while the Saharan aerosols are characterized by ~100% dust Fe (Fig. 3). This clearly demonstrates the dominance of anthropogenic Fe to some open-ocean regions of the North Atlantic, and also how δ^56^Fe can be used to place direct constraints on the two Fe sources in aerosols.

**Fig. 3:**
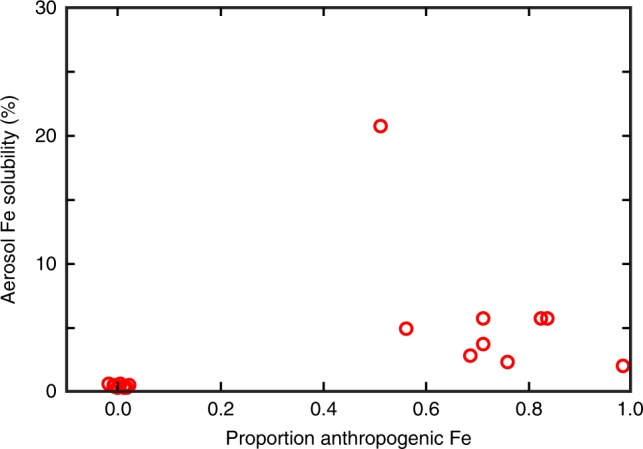
Water-soluble GA03 aerosol Fe from anthropogenic sources. Proportions of Fe from anthropogenic sources were calculated using a simple two-component isotope mixing equation, and hypothesized δ^56^Fe end members of 0.09‰ for dust and −1.6‰ for anthropogenic Fe (see main text for details)

**Fig. 4:**
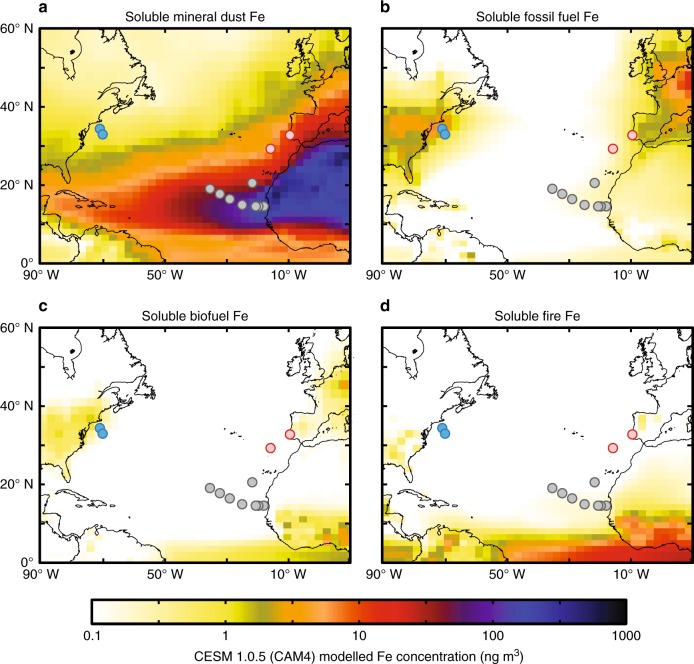
Modeled aerosol Fe for the North Atlantic. CAM4 baseline model soluble aerosol Fe (combined October + November deposition) of aerosol Fe concentration for crustal mineral dust, fossil fuel, biofuels, and fire, with sampling locations in this study shown. Panels show soluble aerosol Fe concentration output for **a** mineral dust, **b** fossil fuel, **c** biofuel, and **d** fire from the Community Earth System Model (CESM 1.0.5) with the Community Atmosphere Model (CAM4) bulk aerosol model, using intermediate complexity soluble atmospheric processing (baseline CAM4 scenario)^49,50^. GA03 sampling locations are color coded as in Fig. 1

While the pattern of isotopically light soluble aerosol δ^56^Fe observed in the North Atlantic in this study provides evidence for an important anthropogenic aerosol Fe source, it does not identify whether the anthropogenic Fe comes from fossil fuel or biofuel combustion, as previous studies have attributed the observation of isotopically light Fe to either one of these sources. However, comparison with aerosol deposition models can help to answer this question. To inform this, we considered model deposition output from the Community Earth System Model (CESM 1.0.5) with the Community Atmosphere Model (CAM4) bulk aerosol model, which has recently been adapted to include an intermediate complexity soluble Fe atmospheric processing scheme (denoted here as the baseline scenario)^49,50^. This version of the CAM4 model simulates both mineral dust Fe and combustion (fossil fuel, biofuels, and fire) Fe emissions, with transport and atmospheric processing to simulate soluble Fe deposition for each type of aerosol (Fig. 4). As can be seen from Fig. 4, the spatial patterns during the time of GA03 aerosol collection (October–November) are most consistent with the light δ^56^Fe resulting from fossil fuel combustion sources, which are the dominant source of anthropogenic aerosols in the North Atlantic at this time of year, and not biomass burning (i.e., fires are not prevalent in Northern Hemisphere mid-latitude during winter).

**Fig. 5:**
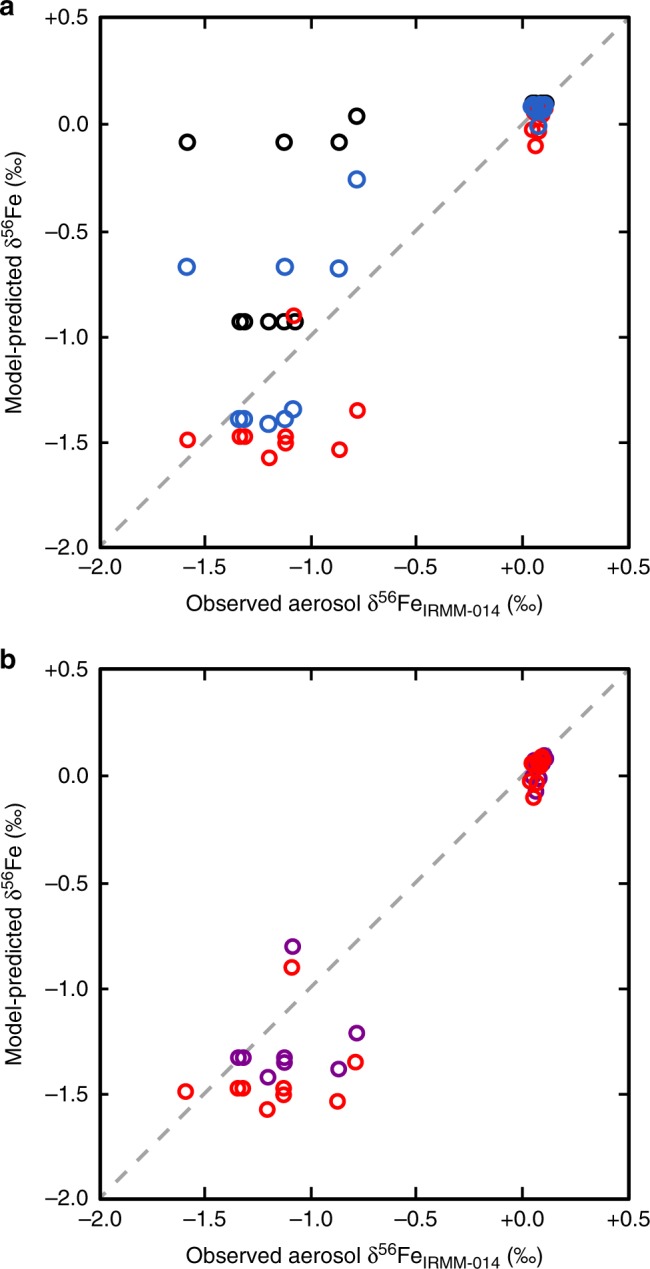
Comparison of GA03 observations with model predictions. Black circles denote output from CAM4 baseline model scenario of Scanza et al.^49^ with monthly average output and end members of +0.09‰ for dust and −1.6‰ for anthropogenic Fe, and shows poor agreement. In **a**, agreement is improved by changing model to use daily mineral dust to capture dust storms and reduced dust source solubilization (from 25 to 10%) (blue circles). Agreement is further improved in **a** by using 5× anthropogenic emissions, to generate an isotope-refined model scenario (CAM4_isotope-refined_ scenario) which best matches GA03 observations (red circles). The anthropogenic endmember was also optimized in **b** to provide best fit between model and observations (see main text; red points are with an anthropogenic δ^56^Fe endmember of −1.6, purple circles are with an optimized anthropogenic δ^56^Fe endmember of −1.4‰)

As well as providing spatial information about which source may be responsible for the isotopically light Fe, deposition model output provides the opportunity to test how well models are capturing discrete aerosol observations, and whether the hypothesis linking isotopically light Fe to anthropogenic sources is consistent with current model scenarios. Here, we directly compared output from the baseline CAM4 deposition model scenario^49^ for the North Atlantic with GA03 aerosol observations, in terms of both Fe solubility and a predicted aerosol δ^56^Fe at the location and time the GA03 samples were collected. While the baseline CAM4 model produced similar solubilities (4–8%) to GA03 measurements (mostly 2–6%) for the North American and European aerosols, CAM4 overestimated observations for Saharan Aerosols (median 2% vs 0.4%), resulting in a poor correlation between model-predicted and observed soluble δ^56^Fe of aerosols, when an δ^56^Fe_anthropogenic_ endmember of −1.6‰ was used (Fig. 5). It is worth noting that this correlation cannot be improved simply by using a lighter δ^56^Fe_anthropogenic_ such as −3‰. However, initial model-observation comparisons were based on a monthly mean model deposition output which was compared with the sub-weekly (often daily) observations. However, the monthly mean will likely miss the details of discrete dust storms which occur on a sub-monthly time frame. Increasing the model temporal resolution to daily mean model output allowed for a more direct comparison with the days of collection and hence improved the model-observation comparison (reduced median solubility from 2.0 to 1.4%). However, this was still higher than GA03 observations.

A reduction in the solubility of readily released Fe (the Fe assumed soluble at emission) in mineral dust at source from 25 to 10% within the model also improved the fit to solubility data (median of 0.81%), improving the δ^56^Fe predictive power compared with observations (Fig. 5a). However, even this modeling scenario still failed to capture the lightest δ^56^Fe values, which suggested that the magnitude of the soluble anthropogenic aerosol was being underestimated. Recent work based on magnetite observations in China^27^, has suggested that anthropogenic aerosol Fe emission are currently underestimated globally, potentially up to a factor of 8. To test this, we increased combustion Fe emissions (fossil fuel, biofuel, and fire) by 5× here to match the CAM4 NEW scenario of Matsui et al.^27^, to see if this provided a better fit to the observed lighter δ^56^Fe. We found that this 5× increase in anthropogenic emissions, together with the changes to mineral dust outlined above (denoted here as the CAM4_isotope-refined_ scenario) produced a good fit to GA03 observations (Fig. 5a).

Following refinement of the CAM4 model to match GA03 observations in this study using an assumed anthropogenic endmember of −1.6‰, we took the CAM4_isotope-refined_ output and optimized the slope of the trendline of the model observations to as close to 1:1 as possible, simply by varying the anthropogenic δ^56^Fe endmember while keeping the dust δ^56^Fe endmember fixed (Fig. 5b). This exercise, while not influencing the soluble Fe deposition output of the model, may provide a better constraint on the specific isotope endmember for anthropogenic Fe in this region (−1.43‰). While this predicted endmember is slightly heavier than the lightest GA03 observation (−1.58 ± 0.08‰), it is lighter than all but one North American and European GA03 aerosols, and significantly heavier than some of the potential combustion sources that have been described from Japan^34,40^. This variability may reflect variability in regional sources, source signatures or grain size effects in the two basins. For example, combustion products are isotopically heavy (+0.4‰^34^) in the coarser size fractions (>2 μm). Interestingly, coarser-grained heavier anthropogenic Fe might explain the observations of Fe which is heavier-than-crustal in one of our European sample total digests (+0.3‰), but which was not obvious in the corresponding soluble fraction (−0.78‰), and in several previously reported Equatorial Pacific aerosols (+0.3 to +0.4‰^51^). Size-fractionated marine aerosol collection, coupled with soluble δ^56^Fe analysis, while challenging, would therefore help to provide better constraints on the range of anthropogenic δ^56^Fe end members in future studies.

The overall observation of an isotopically light water-soluble Fe phase in North Atlantic aerosols, and the changes made to produce our CAM4_isotope-refined_ scenario for the North Atlantic have implications for both the global ocean Fe isotope budget and, perhaps more importantly, the flux of soluble aerosol Fe to the oceans.

**Fig. 6:**
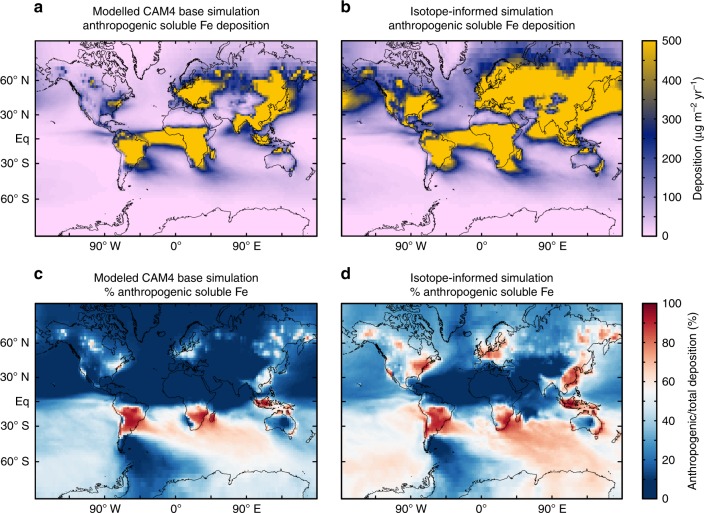
Global impact on modeled deposition of soluble Fe. CAM4_isotope-refined_ scenario compared with baseline CAM4 simulation of Scanza et al.^49^ (see Fig 3; 5× increased anthropogenic Fe, and reducing solubilization of mineral dust at source to 10%). Panels **a** and **c** show the CAM4 base simulation, and **b** and **d** show the CAM4_isotope-refined_ scenario. Anthropogenic soluble Fe deposition was calculated as fossil fuel + biofuel + fire soluble Fe deposition. Percentage of anthropogenic soluble iron was calculated as anthropogenic soluble Fe deposition divided by anthropogenic + mineral dust soluble Fe deposition

Previously, studies have used seawater δ^56^Fe as a tool to trace different sources of Fe to the ocean, assuming that each source has a single unique endmember^6,32,52^. Previous work in the North Atlantic, based on water-column data from the GA03 section, assumed a dust endmember δ^56^Fe of +0.7‰^6^. That study assumed the dust δ^56^Fe endmember was heavier than crustal due equilibrium dissolution of natural mineral dust in concert with strong iron-binding ligands, resulting in an isotopically heavy dissolved Fe pool, over much longer timescale than the instantaneous leaches carried out here^53^. However, data from the present study suggest that a second significant aerosol component should be considered, particularly in regions with high potential deposition fluxes of combustion-derived Fe. Indeed, a continuous background delivery of light δ^56^Fe to the Western North Atlantic could contribute to the observation that the δ^56^Fe of the water column at Bermuda appears to show little variability between season^54^, and is near +0.4‰ throughout the water column^6,55^, lighter than the proposed natural dust endmember.  However, we note this might also be influenced by the recently described process of eddy-driven sediment Fe supply to this region with a near-crustal δ^56^Fe signature^56^. The primary δ^56^Fe signature of deposition to the ocean may thus vary significantly on regional scales as the relative contribution from mineral dust and combustion-derived Fe fluxes changes. This may be especially true in the Pacific Ocean, with the large observed gradient in Asian dust and postulated large changes in the proportion of anthropogenic and natural soluble Fe deposition^26^ (Fig. 6a).

If we apply the isotope-informed changes to the CAM4 baseline model, which are required in the North Atlantic to match observations (5× increase in combustion of Fe and 10× decrease in dust source solubilization) to the baseline CAM4 model^49^ globally to create a new global model scenario (denoted here as CAM4_isotope-refined_), it is clear that soluble anthropogenic Fe deposition dramatically increases at the global scale (Fig. 6a). We calculate that global soluble anthropogenic Fe (fossil fuel, biofuels, and fire) increases with this scenario from 120 to 293 Gg yr^−1^. The effect of this is that anthropogenic Fe deposition becomes more dominant for the soluble Fe aerosol deposition budget at the global scale, especially over large regions of the Southern and Pacific Oceans, but also over Asia, Europe, and North America (Fig. 6b). This finding is consistent with the other previously mentioned recent work using CAM4, which suggests that combustion of Fe must be ~8× greater, in order to reproduce observations of magnetite aerosols, leading to a 50% increase in soluble anthropogenic Fe supply to the Southern Hemisphere and the dominance of combustion as an Fe source to this region^27,57^.

To conclude, we present the first clear evidence that anthropogenic Fe from combustion sources is visible at the basin scale, using δ^56^Fe observations in the North Atlantic. These results suggest that aerosol δ^56^Fe is a promising new tracer for fingerprinting and constraining anthropogenic aerosol Fe sources to the oceans. It is striking to observe anthropogenic aerosol Fe so clearly in a region that is dominated by large deposition fluxes of natural Saharan dust^6,8^, demonstrating the sensitivity of this tracer. Furthermore, we show that aerosol deposition models can be improved by constraining them with δ^56^Fe observations, better capturing the magnitude of anthropogenic Fe fluxes. Here, our model-observation comparison suggests that, in line with other recent observations, anthropogenic combustion sources may be underestimated in current dust model scenarios, while natural dust sources are likely overestimated, meaning that anthropogenic Fe is likely to be a more dominant Fe source to the oceans than previously considered. This is especially true of regions of the ocean which receive much less natural dust^8^ than the North Atlantic and will therefore be proportionately more sensitive to Fe deposition resulting from the distribution and degree of industrial combustion. Near-future increases in anthropogenic Fe supply, such as increased Southern Hemisphere industrialization and increased Northern Hemisphere dust sources may therefore lead to episodic and short-term changes in carbon sequestration which may act to mitigate long-term increases in atmospheric CO_2_ from anthropogenic emissions.

## Methods

### Data availability

All GA03 aerosol δ^56^Fe data from this study are available in Suppl. Data, with GA03 water-soluble and bulk elemental concentrations for this subset of GA03 aerosols. GA03 bulk aerosol Fe data are reproduced from Shelley et al.^43^, GA03 soluble aerosol Fe concentration data are reproduced from Wozniak et al.^44,45^ and Shelley et al.^58^. All GA03 aerosol concentration data are available in the 2017 GEOTRACES Intermediate Data Product^4^.

### Laboratory and analytical methods

Shipboard aerosols were collected on board the R/V Knorr as part of the US GEOTRACES North Atlantic GA03 section cruises in October–November 2010 (USGT10) and November 2011 (USGT11), and sampling details are shown in Supplementary Table 1. Samples were collected onto acid-washed 47 mm Whatman 41 ashless filter discs using a high-volume aerosol sampler (1.2 m^3^ air min^−1^) on the ship’s flying bridge (14 m above sea level), with a procedure designed to avoid contamination from the ship’s stacks, as previously described^43^. On the ship, the filters were leached rapidly with 100 mL of ultrapure water (>18 MΩ.cm; pH 5.5) to release the instantaneously water-soluble phase^59^, which was then filtered through a 0.45 µm pore-size GN-6 cellulose filter, and the leachate acidified to 0.024 M HCl (pH ~1.7) for later (onshore) analysis of elemental concentrations^44^. The remaining filters were stored frozen, prior to onshore processing for determination of total particulate (bulk) concentrations following a HF-HNO_3_ total digestion procedure^43^. Samples were analyzed for trace metal elemental concentrations using an Element II HR-ICP-MS at Florida State University (FSU), and have been the focus of other studies^43–45^.

Full deposition and procedural leaching blanks for total digests and water-soluble samples were 200–800 ng and 3–9 ng (per filter), respectively, based on blank filters deployed on the ship^43^. The means of these blank values corresponds to <0.4% (Saharan) and 4–82% (Euro and North American) of the total digests and <1% (Saharan) and 2–5% (Euro and North American) of the sample for the ultrapure water leaches (see Suppl. Data for % blank contribution). The δ^56^Fe of two total-digested deposition blanks for 2011 were 0.00 ± 0.16‰ and +0.47 ± 0.06‰, while dissolved deposition blanks were of too low concentrations to analyze for δ^56^Fe. We note that the two bulk samples from non-Saharan air masses which were the only total samples with a high calculated blank component (60–80%), were also the only two bulk non-Saharan samples with isotopically heavy δ^56^Fe values. This observation suggests that the primary isotopically light aerosol signature of these two samples was attenuated by a relatively large blank contribution with an isotopically heavy δ^56^Fe values, similar to the blank values mentioned above. As we have insufficient constraints to correct for this blank contribution, these two samples were excluded from the means calculated here.

Of the 39 aerosol samples collected on the GA03 cruises and processed for total or water-soluble elemental concentrations^43,45^, aliquots of the total digests and deionized water leachates from 17 of the samples were processed and analyzed for stable Fe isotope ratios (δ^56^Fe). Samples were chosen based on those which had sufficient Fe for isotopic analysis, and to sample aerosols from North American, Saharan, and European air masses^43^. Second, a subset of the GA03 aerosol-laden filters from North American air (19.87–21.41 N, 51.46–52.47 W) and Saharan air were later leached at the University of Alaska (UA) with either ultrapure water (>18.2 M.cm; 0.08 nmol kg^−1^ Fe) or 0.2 μm filtered surface seawater, following a modification (the entire 250–500 mL volume in one aliquot) of previous leaching procedures^47^. The surface seawater used was collected from the Gulf of Alaska (58.4°N 139.14°W; 04/18/13) using clean techniques and had a Fe concentration of 2.63 nmol kg^−1^ Fe and a δ^56^Fe of −0.09‰ (−0.08 ± 0.03‰, −0.10 ± 0.03‰; 2σ). Due to the relatively high Fe in the seawater (10–20% of the Fe leached; Suppl. Data), and a well-constrained background seawater δ^56^Fe, the seawater-leached δ^56^Fe was corrected using the simple assumption of two-component mixing: δ^56^Fe_dissolved_ = (*f*_BL_ × δ^56^Fe_BL_) + (*f*_SAM_ × δ^56^Fe_SAM_), where BL = background medium, and SAM = aerosol samples (corrected values are shown in Table 1 and both values are shown in Suppl. Data).

Samples were processed for δ^56^Fe using previously published techniques^60^. All work was carried out under ULPA-filtered air, all water was >18.2 MΩ.cm and all reagents were Aristar Ultra^TM^, obtained from VWR International. Briefly, aliquots of either total digests or aerosol leachates were spiked with an ^57^Fe–^58^Fe double spike in a 1:2 sample:spike ratio and then evaporated to dryness, before being purified by AGMP-1 anion-exchange column chemistry. Alaskan seawater and seawater leaches were spiked with Fe double spike in a similar manner and then processed using a Nobias PA1 chelating resin extraction and AGMP-1 purification technique for the determination of δ^56^Fe in seawater^60^. δ^56^Fe ratios were analyzed by Neptune MC-ICPMS at the University of South Carolina and are expressed relative to the IRMM-014 international Fe isotope standard:

$${\mathrm{\delta }}^{56}{\mathrm{Fe}}\,(‰) = \left[ {\frac{{\left( {\frac{{{\,}^{56}{\mathrm{Fe}}}}{{{\,}^{54}{\mathrm{Fe}}}}} \right)_{\text{sample}}}}{{\left( {\frac{{{\,}^{56}{\mathrm{Fe}}}}{{{\,}^{54}{\mathrm{Fe}}}}} \right)_{\text{IRMM} - 014}}} - 1} \right]\, \ast \,1000$$

Each processed aerosol sample was analyzed by MC-ICPMS twice, and mean δ^56^Fe values were calculated. Where two aliquots of the total-digest were processed separately, a mean has been calculated. Sample data in plots are expressed with 2σ internal uncertainty on δ^56^Fe ratios based on the internal analytical error of samples and bracketing standards following our previous work^60^. We estimate 2σ external precision to be 0.05‰ for the water and seawater-soluble aerosol leach samples based on replicate similar analyses of δ^56^Fe in 60 seawater samples^54^ (with duplicates measured over several analytical sessions during a similar time period as these samples), and 0.05‰ for the total-digested aerosol samples (based on duplicate analyses of 22 total-digested Fe samples in this study (with internal errors 0.02–0.04‰) over three analytical sessions, excluding two lower concentration samples which had internal errors >0.1%). We therefore regard these 2σ external error values as a more conservative estimate of uncertainty when larger than 2σ internal error.

### Anthropogenic endmember

There are less available constraints on the anthropogenic endmember compared with Saharan dust. However, we used data from this study and the literature to choose the most representative value. The δ^56^Fe endmember cannot be heavier than −1.6‰, because using a heavier value than this would lead to f >1 for some samples in this dataset. The lightest potential value for the endmember can be constrained by published observation of fine-fraction bulk aerosol from Hiroshima, Japan (−2.01 to −0.56‰^34^), from marine aerosols in the North West Pacific (−1.17 to −1.72‰^34^) and from a road tunnel near Hiroshima (−3.1 to +0.3‰^40^). In the latter near-source case, however, δ^56^Fe composition was particle size dependent, with only the smallest size fraction (0.2 μm) at −3‰, 0.5 μm at −1.5‰, and larger particles at heavier δ^56^Fe values^40^. Although published size-fractionated marine aerosol information is globally scarce, limited information suggests that most soluble Fe in South Atlantic aerosol is in larger size fractions than 0.2 μm^61,62^, meaning that if North Atlantic aerosols are comparable, a near-source δ^56^Fe −3‰ would likely be too light a choice. Considering the available constraints, we suggest −1.6‰ is most applicable for the anthropogenic endmember in calculations, since this both falls within the range of observations (−1.5 to −2‰), and was directly observed in the GA03 North Atlantic dataset.

### Modeling output

Deposition output for this study was taken from the Community Earth System Model (CESM 1.0.5) with the Community Atmosphere Model (CAM4) bulk aerosol model, using intermediate complexity soluble atmospheric processing, as described previously^49^ (here defined as CAM4 baseline). The model was initially run to give monthly average deposition for October or November as appropriate to the observations and to match the relevant grid square (Fig. 4). The model was then adapted to output daily data in order to properly capture dust storms and Fe solubility (see main text, Fig. 5); collection start day was used to compare with all GA03 samples except 5965, where collection end day was used as the model deposition showed a peak in mineral dust occurring during the period of collection rather than at beginning of collection. The original model parametrization was modified (denoted as CAM4_isotope-refined_) to reduce mineral dust solubilization from 25 to 10%, and to increase anthropogenic combustion emissions by 5× compared with the baseline CAM4 scenario of Scanza et al.^49^ in order to generate Figs. 5 and 6 and conclusions.

## Supplementary information

Description of Additional Supplementary Files

Supplementary Data 1

## Data Availability

All Fe concentration and δ^56^Fe data reported in this study are available as Supplementary Data or are previously published^43–45,58^. All model output and codes will be publicly available either by request to the authors or from http://www.geocornell.edu/eas/PeoplePlaces/Faculty/mahowald/dust/Conwayetal2019/.
