# Supplementary material for: Tracing and constraining anthropogenic aerosol iron fluxes to the North Atlantic Ocean using iron isotopes
Source: Nat Commun. 2019 Jun 14;10:2628. doi: 10.1038/s41467-019-10457-w (PMC6570766; doi:10.1038/s41467-019-10457-w)
Supplement: Supplementary file 1 — Description of Additional Supplementary Files [file 41467_2019_10457_MOESM1_ESM.pdf]

Description of Additional Supplementary Files

File Name: Supplementary Dataset 1
Description: GA03 aerosol Fe isotope data. Dataset includes aerosol samples processed by Florida State University and University of Alaska Fairbanks, and measured at the University of South Carolina.
